# Genetic and Epigenetic Therapies for β-Thalassaemia by Altering the Expression of α-Globin Gene

**DOI:** 10.3389/fgeed.2021.752278

**Published:** 2021-09-30

**Authors:** Sachith Mettananda

**Affiliations:** ^1^ Department of Paediatrics, Faculty of Medicine, University of Kelaniya, Ragama, Sri Lanka; ^2^ University Paediatrics Unit, Colombo North Teaching Hospital, Ragama, Sri Lanka

**Keywords:** genome editing, CRISPR/Cas9, vorinostat, IOX1, β-thalassaemia, α-globin, α-thalassaemia

## Abstract

β-Thalassaemia is caused by over 300 mutations in and around the β-globin gene that lead to impaired synthesis of β-globin. The expression of α-globin continues normally, resulting in an excess of α-globin chains within red blood cells and their precursors. These unpaired α-globin chains form unstable α-hemichromes that trigger cascades of events to generate reactive oxygen species, leading to ineffective erythropoiesis and haemolysis in patients with β-thalassaemia. The clinical genetic data reported over several decades have demonstrated how the coinheritance of α-thalassaemia ameliorates the disease phenotype of β-thalassaemia. Thus, it is evident that down-regulation of the α-globin gene expression in patients with β-thalassaemia could ameliorate or even cure β-thalassaemia. Over the last few years, significant progress has been made in utilising this pathway to devise a cure for β-thalassaemia. Most research has been done to alter the epigenetic landscape of the α-globin locus or the well-characterised distant enhancers of α-globin. In vitro, pre-clinical studies on primary human erythroid cells have unveiled inhibition of histone lysine demethylation and histone deacetylation as potential targets to achieve selective downregulation of α-globin through epigenetic drug targeting. CRISPR based genome editing has been successfully used in vitro to mutate α-globin genes or enhancers of α-goblin to achieve clinically significant knockdowns of α-globin to the levels beneficial for patients with β-thalassaemia. This review summarises the current knowledge on the regulation of human α-globin genes and the clinical genetic data supporting the pathway of targeting α-globin as a treatment for β-thalassaemia. It also presents the progress of epigenetic drug and genome editing approaches currently in development to treat β-thalassaemia.

## Introduction

β-Thalassaemia is one of the most common monogenic diseases affecting the red blood cells (RBC) (Taher et al., 2018). It has been historically more prevalent in the tropical regions extending from the Mediterranean and the middle east to south and southeast Asia (Mettananda et al., 2020a). However, due to recent migration patterns, it now affects every country in the world (Weatherall, 2011). Most patients with β-thalassaemia receive only supportive treatment; therefore, they die prematurely and have a poor quality of life (Mettananda et al., 2019a; Mettananda et al., 2019b; Mettananda et al., 2020b).

Allogeneic haematopoietic stem cell (HSC) transplantation (HSCT) was the only available cure for β-thalassaemia for many decades (Baronciani et al., 2016; Mettananda, 2018a). However, since obtaining the regulatory approval from the European Medicines Agency in June 2019, *ex vivo* gene therapy using BB305 lentiviral vector containing a transgene encoding β^A−T87Q^-globin is available as a cure for transfusion-dependent β-thalassemia patients older than 12 years (Thompson et al., 2018; Harrison, 2019). In fact, β-thalassaemia is one of the first diseases for which the gene therapy was approved. Despite this progress, several challenges remain that limit gene therapy as a cure for β-thalassaemia. They are; lack of efficacy in patients with severe β^0^/β^0^-thalassaemia phenotype, poor efficiency of gene expression, high cost and potential risks, including insertional oncogenesis, generation of replication-competent lentiviruses and off-target effects (Soni, 2020).

To overcome the challenges of HSCT and traditional gene therapy, several new approaches to treat β-thalassaemia are being studied. Inhibition of ineffective erythropoiesis by activin IIB receptor-ligand trap luspatercept, induction of fetal haemoglobin by hydroxyurea, upregulation of γ-globin by epigenetic drugs and genome editing of suppressors of γ-globin are notable examples (Piga et al., 2019; Cappellini et al., 2020; Yasara et al., 2020; Frangoul et al., 2021; Yasara et al., 2021). An alternative pathway that has shown promise is silencing of α-globin. This review aims to provide the rationale and summarise the progress of epigenetic and genome editing research on targeting α-globin as a cure for β-thalassaemia.

### The Role of Free α-Globin in the Pathophysiology of β-Thalassaemia

β-Thalassaemia is caused by over 300 autosomal recessively inherited mutations in and around the β-globin gene in chromosome 11 (Taher et al., 2021). Homozygous or compound heterozygous inheritance of these mutations leads to β-thalassaemia major, a transfusion-dependent form of thalassaemia (Mettananda and Higgs, 2018). Compound heterozygous inheritance of one β-thalassaemia mutation with the haemoglobin E mutation results in haemoglobin E β-thalassaemia, another common variant of β-thalassaemia (Fucharoen and Weatherall, 2012).

**FIGURE 1:**
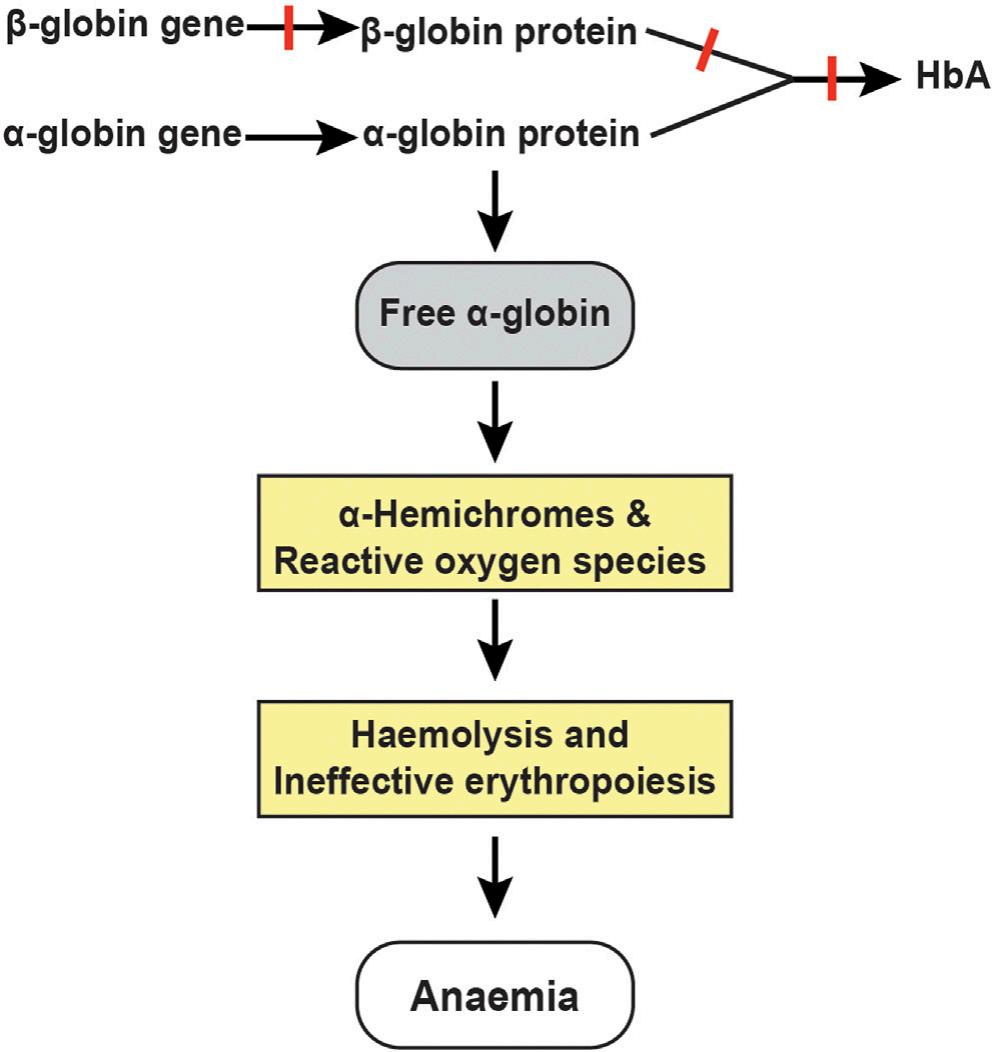
Pathophysiology of β-thalassemia. Absent or reduced β-globin production leads to an unbalanced excess of α-globin chains, which trigger a cascade of events through the generation of α-hemichromes and reactive oxygen species resulting in haemolysis and ineffective erythropoiesis.

Adult haemoglobin is a tetramer of two α- and two β-globin chains. In normal human erythroid cells, a tight balance between the α-globin and β-like globin (β-globin and γ-globin) chains is maintained (Weatherall, 2010). Disequilibrium of the globin chains due to reduced or absent synthesis of β-globin chains in erythroid cells is the primary pathophysiology of β-thalassaemia (Schrier, 2002). Consequently, free α-globin chains that do not have adequate β-like globin chains to pair with, form insoluble α-hemichromes that trigger a cascade of events through the generation of reactive oxygen species leading to premature destruction of erythroid cells (Figure 1); (Schrier et al., 2003). Thus, excess free α-globin chains are the main mediators of ineffective erythropoiesis and haemolysis in β-thalassaemia (Allen et al., 2021).

### Co-Inheritance of α-Thalassaemia Ameliorates the Severity of β-Thalassaemia

Several clinical genetic studies done among patients with β-thalassaemia major and haemoglobin E β-thalassaemia have conclusively identified coinheritance of α-thalassaemia as a favourable genetic modifier that ameliorates the disease phenotype of β-thalassaemia (Mettananda et al., 2015). When coinherited, deletion of one or two (out of four) α-globin genes reduces the free α-globin pool and minimises the haemolysis and ineffective erythropoiesis (Premawardhena et al., 2005). Thus, a patient who would otherwise develop transfusion-dependent thalassaemia will transform into a less severe phenotype of non-transfusion dependent thalassaemia. Specifically, deletion of two α-globin genes (--/αα or -α/-α) reduces the severity in most patients with β-thalassaemia, whereas deletion of a single α-globin gene (-α/αα) ameliorates the disease phenotype in all patients except those with the most severe β^0^/β^0^-thalassaemia phenotype (Ho et al., 1998; Galanello et al., 2009; Mettananda et al., 2015). Also, the coinheritance of α-thalassaemia improves the RBC indices and haemoglobin levels among individuals with heterozygous β-thalassaemia trait (Mettananda et al., 2018a). In summary, a 25–50% reduction in α-globin chain output in RBC could significantly lessen the severity of β-thalassaemia (Mettananda et al., 2015).

### Regulation of Human α-Globin Gene Expression

The human α-globin genes are in the short arm of chromosome 16 (16p13.3), approximately 150 kb from the telomere (Hughes et al., 2005). Two α-globin genes, *HBA1* and *HBA2*, are in each chromosome; therefore, each human cell have four copies of α-globin genes. The expression of α-globin genes is regulated by a complex interplay between gene promoters, enhancers, boundary elements, transcription factors and epigenetic modifications of the associated chromatin (Mettananda et al., 2016; Oudelaar and Higgs, 2021). It is believed that during transcription, enhancers and promoters are brought into close physical proximity to each other by chromatin looping (Mettananda et al., 2016).

**FIGURE 2:**
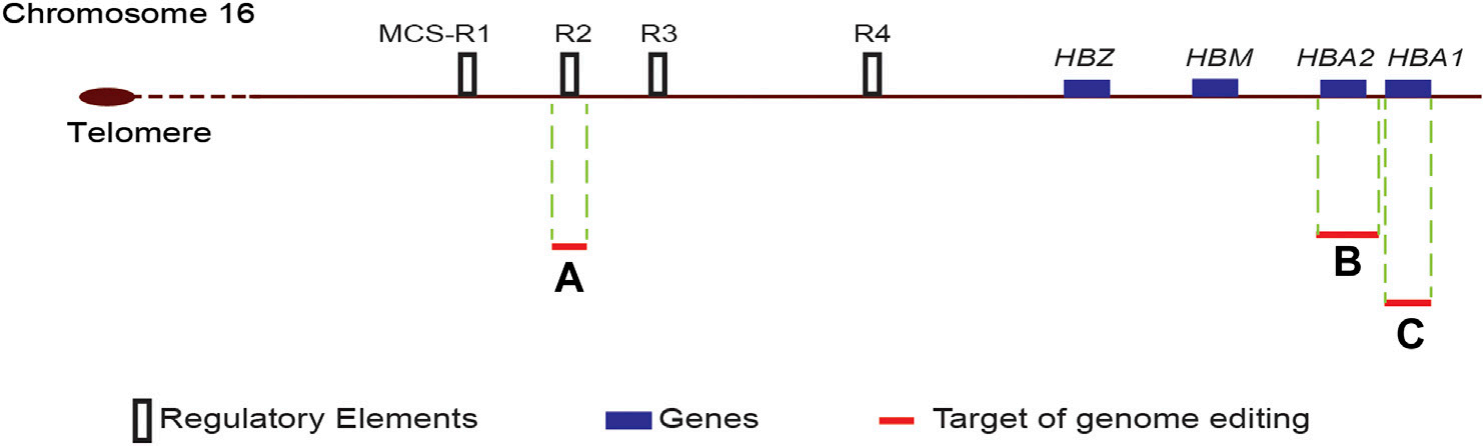
Schematic diagram of the α-globin locus. The human α-globin locus, which consists of two α-globin genes (*HBA1* and *HBA2*), μ-globin (*HBM*) and ζ-globin (*HBZ*), is located in the short arm of chromosome 16. The four distant enhancers, multispecies conserved sequence (MCS) R1 to R4, are located upstream of the ζ-globin gene. Target sites for genome editing are indicated by red lines, and the adjacent letter refers to the reference publication. A-(Mettananda et al., 2017b); B-(Pavani et al., 2021); C-(Cromer et al., 2021).

Several *in vitro*, transgenic mice and translational research studies have identified four distant enhancers designated as multispecies conserved sequence (MCS) R1 to R4 located 10–48 kb upstream to the gene locus, critical for the expression of α-globin genes (Figure 2). Of these enhancers, the MCS-R2 located 40 kb upstream of the α-globin gene has been shown as the most critical enhancer influencing the expression of α-globin genes. In transgenic humanised mice, deletions involving the MCS-R2 region led to a marked reduction of the α-globin expression to very low levels (Wallace et al., 2007; Farashi and Harteveld, 2018). A rare naturally occurring homozygous deletion of the MCS-R2 region resulted in severe α-thalassaemia despite having all four α-globin genes intact (Coelho et al., 2010).

### Epigenetic Drug Targeting of α-Globin

**FIGURE 3:**
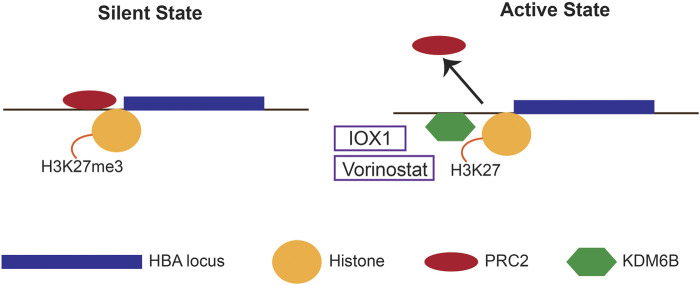
Epigenetic mechanisms regulating α-globin expression. In the silent state, the human α-globin locus is silenced through binding of the Polycomb repressive complex 2 (PRC2) and its associated repressive chromatin signature H3K27me3. In the active state, the PRC2 is detached, and histone lysine demethylase 6B (KDM6B) is recruited to facilitate the removal of the H3K27me3. Epigenetic drugs that down-regulate α-globin expression (IOX1 and vorinostat) inhibits KDM6B.

Epigenetic modifications play a pivotal role in regulating the α-globin gene expression (Mettananda, 2018b). The human α-globin gene cluster is in a gene dense, early replicating, open chromatin region of the genome, therefore, requires active silencing through epigenetic mechanisms (Figure 3); (De Gobbi et al., 2007). The α-globin genes in non-erythroid cells are silenced through binding of the Polycomb repressive complex 2 (PRC2), which is associated with histone methyltransferase that increases the repressive chromatin signature Histone 3 Lysine 27 trimethylation (H3K27me3) (Garrick et al., 2008). In erythroid cells, when α-globin gene is activated, PRC2 is displaced, and the H3K27me3 chromatin signatures are removed by histone lysine demethylase (KDM) 6B (Vernimmen et al., 2011).

Altering these epigenetic signatures using epigenetic inhibitor drugs to silence the α-globin has been attempted. Inhibition of histone lysine demethylation by a novel molecule IOX1 has shown to downregulate the expression of α-globin *in vitro* in primary human erythroid cells (Mettananda et al., 2017a). IOX1 is a pan-histone demethylase inhibitor that inhibits a number of KDM enzymes at various concentrations. Thus, inhibition of KDM6B is believed to be the mechanism of action of IOX1 in human erythroid cells.

Similarly, histone deacetylase (HDAC) inhibitor vorinostat has been shown to silence α-globin whilst upregulating the expression of γ-globin in human erythroid cells (Mettananda et al., 2019c). Vorinostat is a hydroxamic acid-group pan-HDAC inhibitor that causes significant upregulation of γ-globin similar to other HDAC inhibitors (Okam et al., 2015). Although the exact mechanism of downregulation of α-globin by vorinostat is uncertain, it may act through the KDM6B. In summary, both IOX1 and vorinostat have shown considerable promise in *in vitro* pre-clinical studies in human erythroid cells. However, their clinical usefulness is yet to be tested in clinical trials.

### Genome Editing Therapies for β-Thalassaemia

β-Thalassaemia has several salient features for it to become a promising candidate disease for the development of genome editing therapies (Doudna, 2020). Firstly, the genetic basis and the molecular pathology of β-thalassaemia is well understood (Taher et al., 2021). Secondly, the genome edits are only needed to be performed in HSC, which can easily be mobilised from patients for *ex vivo* manipulations. Thirdly, the technologies for mobilisation and subsequent transplantation of HSCs are highly advanced at present due to the long-standing clinical use of HSCT for multiple indications (Ferrari et al., 2021). Therefore, β-thalassaemia has been at the forefront to bring genome editing therapies to the bedside. Any genome editing treatment aiming to cure β-thalassaemia shall involve mobilisation and collection of HSCs, introducing the desired genome edits *ex vivo* and transplanting back to the patients after myeloablative conditioning (Mettananda, 2020).

Genome editing to upregulate γ-globin expression with a view of increasing HbF synthesis has been successful in clinical trials (Demirci et al., 2020a). The scientific rationale behind this is to increase the production of γ-globin chains, which would then mop-up the excess of α-globin chains to reduce free α-globin chains, thus improving the α/β-like globin disequilibrium in RBCs (Bauer et al., 2012). *XmnI* polymorphism (rs7482144) located within the promoter of γ-globin gene, *HBS1L-MYB* intergenic region on chromosome 6q23 and *BCL11A* gene located on chromosome 2p16 are the genomic targets to upregulate γ-globin (Menzel et al., 2007; Thein et al., 2007; Sankaran et al., 2008; Demirci et al., 2020b; Zeng et al., 2020).

Therapeutic disruption of the expression of B-cell lymphoma/leukaemia 11A protein (BCL11A), which is a critical transcription factor involved in postnatal silencing of γ-globin, has been the target of recent clinical trials (Psatha et al., 2018; Canver et al., 2015). One of these clinical trials (ClinicalTrials.gov Identifier NCT03432364) utilised zinc finger nucleases (ZFN) as the genome editing tool. In this trial, CD34^+^ HSCs were transfected with mRNA encoding ZFNs, which has binding sites for the GATA-binding region of the enhancer of *BCL11A*. Preliminary results of this trial reports elevation of HbF in two patients after receiving infusions of autologous genome edited CD34^+^ HSC product ST-400. However, at least one patient failed to remain transfusion independent (Smith et al., 2019).

In contrast, another clinical trial (ClinicalTrials.gov Identifier NCT03655678) used CRISPR/Cas9 gene editing to disrupt an erythroid-specific enhancer region of *BCL11A*. In this trial, a female patient with transfusion-dependent β-thalassaemia who received the autologous genome edited CD34^+^ HSC product CTX001 showed sustained elevations of HbF and remained transfusion independent (last reviewed after 18 months post-transplant) (Frangoul et al., 2021). In addition to these clinical trials, the disruption of a γ-globin gene promoter motif bound by BCL11A by CRISPR/Cas9 has induced HbF to potentially therapeutic levels in *in vitro* studies (Métais et al., 2019).

Furthermore, CRISPR based genome editing has been used to correct some β-thalassaemia mutations *in vitro*. Allelic disruption of aberrant splice sites of IVS1-110G > A and IVS2-654C > T mutations and repair of CD39 (CAG > TAG) mutation using a single-strand donor DNA template have shown promising results in pre-clinical studies (Xu et al., 2019; Cosenza et al., 2021).

### Genome Editing of α-Globin Genes

Genome editing of α-globin gene to improve the α/β globin imbalance is a promising alternative strategy to treat β-thalassaemia. This strategy aims to decrease the α-globin output by 25–50% to reproduce the beneficial natural effect of α-thalassaemia trait on transfusion-dependent β-thalassaemia. CRISPR/Cas9 genome editing to down-regulate α-globin by deleting one of the two α-globin genes (*HBA2*) to recreate an α-thalassemia trait was reported in a recent *in vitro* study (Pavani et al., 2021). In this study, the investigators designed a single guide RNA (sgRNA) to target the 5′UTR of *HBA1* and *HBA2* genes, thus removing the *HBA2* gene (Figure 2). Erythroid cells generated from a β-thalassaemia HSC line (HUDEP-2 β^0^) transfected with ribonucleoprotein containing this sgRNA resulted in a dramatic reduction of α-globin messenger RNA and a significant decrease in α-globin precipitates in erythroid cells. Transfection of HSC of β^+^-thalassaemia with these ribonucleoproteins and differentiation of edited HSC into erythroid cells resulted in correction of the pathologically high α/β-globin ratios.

### Genome Editing of α-Globin Enhancers

Another approach to reduce α-globin chain synthesis in human erythroid cells is to genome edit the MCS-R2 critical α-globin enhancer. As postulated by natural mutations involving the MCS-R2 enhancer, a single allelic genome editing of the MCS-R2 decreases the α-globin synthesis by up to 50%, whereas homozygous biallelic mutation would reduce the α-globin synthesis to a level seen in patients with haemoglobin H disease. A recent *in vitro* study reported successful replication of natural mutations by creating a targeted deletion of the MCS-R2 enhancer using CRISPR/Cas9 genome editing in primary human erythroid cells [(Mettananda et al., 2017b), (Mettananda et al., 2018b)]. In this study, the erythroid cells differentiated from genome edited CD34^+^ HSCs resulted in over 60% knockdown of α-globin chain synthesis following the monoallelic deletion of the MCS-R2 enhancer. Biallelic deletion of the enhancer resulted in a pronounced (∼90%) knockdown of α-globin chain synthesis. The deletion of the MCS-R2 enhancer in erythroid cells of patients with β-thalassaemia could restore the α/β-globin chain imbalance to normal levels. Furthermore, multi-lineage engraftment of genome edited HSC was demonstrated in secondary serial xenotransplantation assays to confirm editing of long-term repopulating HSCs.

### Synergistic Genome Editing

Several genome editing approaches include insertion of β-globin transgene, upregulation of γ-globin and downregulation of α-globin are beneficial to restore the globin balance in β-thalassaemia. Therefore, these approaches could be used in combination to improve the disease severity of patients. In fact, a recent *in vitro* study used CRISPR/Cas9 genome editing to delete one of the two α-globin genes and to integrate a β-globin transgene (β^AS3^) under the control of the endogenous *HBA* promoter (Pavani et al., 2021). Co-transfection of HSC with this β-globin transgene and ribonucleoprotein containing sgRNA designed to delete the *HBA2* gene resulted in an improvement of α/β globin chain to near-normal levels in erythroid cells of patients with β^+^-thalassaemia and β^0^-thalassaemia. Another recent study demonstrated successful use of the combined Cas9/AAV6 genome editing method to replace the endogenous *HBA1* gene with a full-length *HBB* transgene (Cromer et al., 2021). Although the genome editing frequency was below the desired levels, erythroid cells differentiated from edited β-thalassaemia patient HSCs demonstrated normalisation of α/β-globin mRNA and protein ratios and restoration of functional adult haemoglobin tetramers.

Similarly, various combinations of gene therapy, genome editing and other genetic technologies could synergistically treat β-thalassaemia. A recent study demonstrated the use of a modified vector comprised of the standard β^A−T87Q^-globin transgene and an intron-embedded miR-30-based short hairpin RNA designed to target the α2-globin mRNA expression (Nualkaew et al., 2021). Use of this vector showed 1.7-fold greater potency to improve α/β-globin ratios resulting in a greater therapeutic efficacy for β-thalassaemia. Also, genome editing approaches that target downregulation of α-globin can be combined with genome editing approaches of HbF induction to have additive effects. These methods can mitigate the lack of efficacy of individual systems to achieve a precise balance of α to β-like globin chain ratio in RBCs.

### Future Challenges

Although genetic and epigenetic therapies have shown considerable promise, several challenges still limit their ability to develop into standard clinical care (Dong et al., 2013). All genome editing approaches would require the process of HSC transplantation (High and Roncarolo, 2019). Although the risk of graft *vs.* host disease and long-term immunosuppression is eliminated, patients undergoing genome editing still need to undergo myeloablative conditioning before the autologous HSC transplantation. Also, these therapies are technologically demanding thus, are associated with very high costs (Ferrari et al., 2021). Therefore, they would not be available to patients in developing countries who need these treatments most.

Genotoxicity due to undesired DNA modifications is a significant challenge of genome editing. Although most of these changes occur as non-specific off-target effects, a recent study has shown that chromothripsis, a catastrophic genomic rearrangement of chromosomes in a haphazard order, could occur due to on-target effects of genome editing (Leibowitz et al., 2021). Other reported genotoxicity effects of CRISPR/Cas9 genome editing include large-scale DNA deletions or inversions, activation of proto-oncogenes and induction of large chromosomal truncations (Haapaniemi et al., 2018; Kosicki et al., 2018; Cullot et al., 2019). Although newer systems that include high-fidelity Cas9 nucleases and base or prime editing approaches minimises the risk of off-target effects, none of the genome editing tools can be considered hazard free at present (Komor et al., 2016; Anzalone et al., 2019; Magrin et al., 2019).

Another limitation inherent to the current approach is difficulty in titrating the α-globin knockdown to the desirable level. Studies have shown that a 25–50% reduction in α-globin output is the most desirable for optimal α/β globin balance and to ameliorate β-thalassaemia (Mettananda et al., 2015). Too much knockdown will lead to lack of α-globin production and subsequent worsening of anaemia (Mettananda, 2017). Therefore, all genome editing and epigenetic therapies on development towards curing β-thalassaemia by silencing α-globin has a very narrow therapeutic window.

## Conclusion

After several years of optimisation, genome editing for β-thalassaemia has reached clinical trials. The first two clinical trials that target BCL11A to upregulate γ-globin have shown promising results. Considering the pivotal role of excess α-globin and disequilibrium of α/β-globin chain balance in the pathogenesis of β-thalassaemia and the ameliorating effect of coinheritance of α-thalassaemia on β-thalassaemia, downregulating the α-globin expression is considered a potential therapeutic pathway to treat β-thalassaemia. Downregulation of α-globin by genome editing of one of the α-globin genes or their enhancers have shown to improve the α/β-globin imbalance in erythroid cells of β-thalassaemia patients *in vitro*. Also, silencing α-globin can be combined with approaches to insert β-globin transgene or induce HbF to have synergistic effects.
